# Failure to expand the motor unit size to compensate for declining motor unit numbers distinguishes sarcopenic from non‐sarcopenic older men

**DOI:** 10.1113/JP275520

**Published:** 2018-03-23

**Authors:** M. Piasecki, A. Ireland, J. Piasecki, D. W. Stashuk, A. Swiecicka, M. K. Rutter, D. A. Jones, J. S. McPhee

**Affiliations:** ^1^ School of Healthcare Science Manchester Metropolitan University Manchester M15GD UK; ^2^ Department of Systems Design Engineering University of Waterloo Ontario N2L 3G1 Canada; ^3^ Andrology Research Unit, Cardiovascular, Metabolic and Nutritional Sciences Domain, Faculty of Biology, Medicine and Health University of Manchester Manchester UK; ^4^ Manchester Diabetes Centre, Central Manchester University Hospitals NHS Foundation Trust Manchester Academic Health Science Centre Manchester UK

**Keywords:** sarcopenia, muscle, motor unit, denervation, electromyography

## Abstract

**Key points:**

- The age‐related loss of muscle mass is related to the loss of innervating motor neurons and denervation of muscle fibres.
- Not all denervated muscle fibres are degraded; some may be reinnervated by an adjacent surviving neuron, which expands the innervating motor unit proportional to the numbers of fibres rescued.
- Enlarged motor units have larger motor unit potentials when measured using electrophysiological techniques.
- We recorded much larger motor unit potentials in relatively healthy older men compared to young men, but the older men with the smallest muscles (sarcopenia) had smaller motor unit potentials than healthy older men.
- These findings suggest that healthy older men reinnervate large numbers of muscle fibres to compensate for declining motor neuron numbers, but a failure to do so contributes to muscle loss in sarcopenic men.

**Abstract:**

Sarcopenia results from the progressive loss of skeletal muscle mass and reduced function in older age. It is likely to be associated with the well‐documented reduction of motor unit numbers innervating limb muscles and the increase in size of surviving motor units via reinnervation of denervated fibres. However, no evidence exists to confirm the extent of motor unit remodelling in sarcopenic individuals. The aim of the present study was to compare motor unit size and number between young (*n* = 48), non‐sarcopenic old (*n* = 13), pre‐sarcopenic (*n* = 53) and sarcopenic (*n* = 29) men. Motor unit potentials (MUPs) were isolated from intramuscular and surface EMG recordings. The motor unit numbers were reduced in all groups of old compared with young men (all *P* < 0.001). MUPs were higher in non‐sarcopenic and pre‐sarcopenic men compared with young men (*P* = 0.039 and 0.001 respectively), but not in the vastus lateralis of sarcopenic old (*P* = 0.485). The results suggest that extensive motor unit remodelling occurs relatively early during ageing, exceeds the loss of muscle mass and precedes sarcopenia. Reinnervation of denervated muscle fibres probably expands the motor unit size in the non‐sarcopenic and pre‐sarcopenic old, but not in the sarcopenic old. These findings suggest that a failure to expand the motor unit size distinguishes sarcopenic from pre‐sarcopenic muscles.

## Key points

- The age‐related loss of muscle mass is related to the loss of innervating motor neurons and denervation of muscle fibres.
- Not all denervated muscle fibres are degraded; some may be reinnervated by an adjacent surviving neuron, which expands the innervating motor unit proportional to the numbers of fibres rescued.
- Enlarged motor units have larger motor unit potentials when measured using electrophysiological techniques.
- We recorded much larger motor unit potentials in relatively healthy older men compared to young men, but the older men with the smallest muscles (sarcopenia) had smaller motor unit potentials than healthy older men.
- These findings suggest that healthy older men reinnervate large numbers of muscle fibres to compensate for declining motor neuron numbers, but a failure to do so contributes to muscle loss in sarcopenic men.

## Introduction

Low skeletal muscle mass and function in old age, known as sarcopenia, is widely recognised as a serious and independent condition of ageing (Cao & Morley, 2016). An estimated 10–20% of people aged over 65 years have sarcopenia, which is projected to equate to 20–30 million Europeans by 2045 (Ethgen *et al*. 2017).

The progressive loss of muscle mass with increasing age is due to both atrophy and loss of muscle fibres (Lexell *et al*. 1988). Fibre atrophy can be overcome, at least in part, by physical rehabilitation (Doherty, 2003; Brook *et al*. 2016) and pharmaceutical interventions (Dennison *et al*. 2017) but these treatments do nothing to recover lost fibres. These age‐related changes affecting skeletal muscles are likely to be related to declining numbers of motor units (Piasecki *et al*. 2015). A motor unit includes a single alpha motor neuron and all of the muscle fibres it innervates. Anatomical counts estimate 35,000 motor neurons of the upper limbs (Gesslbauer *et al*. 2017) and around 60,000 of the lower limbs (Tomlinson & Irving, 1977), with each innervating hundreds or thousands of muscle fibres (Feinstein *et al*. 1955; Gath & Stålberg, 1981). If a motor neuron is impaired or degraded during ageing, its muscle fibres may lose their innervation and will be vulnerable to apoptosis.

Early indications that motor unit numbers decline with increasing age in humans came from direct counts of neuron cell bodies in the anterior horn of the spinal cord in autopsy samples (Kawamura *et al*. 1977; Tomlinson & Irving, 1977). At around the same time, *in vivo* EMG techniques also revealed decreasing numbers of motor units in a small foot muscle with increasing age (McComas *et al*. 1971). This has since been confirmed for other limb muscles using modern enhanced decomposition EMG techniques (for reviews see Gooch *et al*. 2014; Piasecki *et al*. 2015).

The available evidence not only points towards fewer, but also larger motor units in older adults compared with young (Stalberg & Fawcett, 1982; McNeil *et al*. 2005; Power *et al*. 2012; Hourigan *et al*. 2015; Piasecki *et al*. 2016a,b). Larger motor units are thought to result from branching of nearby motor neurons to reinnervate recently denervated muscle fibres (Luff, 1998; Deschenes, 2011; Hepple & Rice, 2016). In this case, the relative success of reinnervation processes would be expected to determine the extent of muscle atrophy and the progression of sarcopenia.

Despite considerable interest, surprisingly little is known about how motor unit remodelling associates with sarcopenia. Most previous studies did not assess the sarcopenia status of participants, or they included small sample sizes and examined the tibialis anterior, which is relatively well preserved with ageing compared with larger quadriceps muscles (Abe *et al*. 2011; Pannerec *et al*. 2016). Other studies used surface EMG decomposition techniques (Kaya *et al*. 2013; Drey *et al*. 2014), which only sample from the superficial parts of muscles (Muceli *et al*. 2015) and reveal nothing about the size of surviving motor units.

Therefore, the aim of the present study was to compare motor unit size and number between young, non‐sarcopenic, pre‐sarcopenic and sarcopenic men using intramuscular EMG. We hypothesised that (a) there would be a graded decline in motor unit numbers from young through to healthy older men, pre‐sarcopenic and sarcopenic men (i.e. loss of muscle mass would be proportional to the loss of motor units); and (b) there would be a progressive increase in motor unit size from young through to non‐sarcopenic, pre‐sarcopenic and sarcopenic men.

## Methods

### Ethical approval

The study was approved by the University Research Ethics Committee and the NHS Research Ethics Committee (ref: 15NW/0426) and was conducted in accordance with the *Declaration of Helsinki* except for registration in a database. All participants provided written informed consent.

### Participant recruitment

A total of 143 male participants were recruited and included in the study. Inclusion criteria were: male gender, aged 18–40 years or 65–90 years, and living independently. Exclusion criteria included: individuals who lack capacity to consent for the study and comply with the protocol (including those who have a legal guardian); body mass index (BMI) < 18 kg m^−2^ or >35 kg m^−2^; history of cachexia or malnutrition; institutionalised (e.g. living in a nursing home); presence of co‐morbidity [specifically: neurological disorders (stroke resulting in reduced mobility, Parkinson's disease, dementia, motor neuron disease); cancer diagnosis (excluding non‐fatal cancers, e.g. skin cancer, stable prostate cancer, and other stable cancers with a good prognosis); communicable disease such as HIV/AIDS or hepatitis; heart failure (breathless at rest or when walking < 100 m); NYHA III or IV]; permanent pacemaker *in situ* (an exclusion for magnetic resonance scanning only); implantable cardioverter‐defibrillator (ICD) *in situ*; myocardial infarction within the last 6 months, uncontrolled angina, peripheral arterial disease (if this limits function to walking < 100 m), deep vein thrombosis within the last 3 months; severe chronic obstructive pulmonary disease or asthma (causing shortness of breath after a few minutes of walking or with changing clothing (MRC shortness of breath scale grades 4 or 5); coagulation disorder or use of anticoagulants (e.g. warfarin, sinthrome, dabigatran, rivaroxaban, apixaban, low‐molecular‐weight heparin) that could cause excessive bleeding or bruising; lower limb or vertebral fracture within the previous year; hip/knee and/or spinal stenosis surgery during the last 12 months; physical limitation and pain due to conditions that conflicts with study procedures; amputation of part of a lower limb; and non‐fluent speakers of the English language.

### Anthropometry

Body weight (kg) and height (m) were measured and BMI was calculated as weight/height^2^.

Muscle cross sectional area (CSA) and the thickness measured as distance from the superficial to the deep aponeurosis of the quadriceps (QCSA), vastus lateralis (VL) and tibialis anterior (TA) were measured by magnetic resonance imaging (MRI) using a T1‐weighted turbo three‐dimensional sequence on a 0.25‐T G‐Scan imager (Esaote, Genoe, Italy). The scanning coil was positioned over the motor point of the VL and, in separate scans, of the TA. Contiguous 6 mm thick transverse‐plane slices were collected along a 14 cm length with the participant lying rested and supine. Osirix imaging software (OsiriX medical imaging, OsiriX, Atlanta, GA, USA) was used offline to measure the CSA of images by tracing around the quadriceps muscles, and separately the VL muscle for thigh scans, or the TA muscle for lower limb scans following the contour of the aponeurosis. The slice with the highest CSA was recorded as peak CSA (Maden‐Wilkinson *et al*. 2014).

In 14 of the participants (9% of the total sample), MRI was not used due to the presence of medical implants, such as cardiac pacemakers. In these cases, ultrasound was used to measure the muscle thickness and the CSA was then estimated from the regression equation of the very strong linear relationship between muscle thickness and muscle CSA in participants from this study (Pearson product‐moment correlation was *r* = 0.917).

Total body composition was assessed by dual‐energy X‐ray absorptiometry (DXA) (Lunar Prodigy Advance, version EnCore 10.50.086, GE Healthcare, Madison, WI, USA) with the participant lying supine with legs and arms fully extended and wearing light cotton clothing. Appendicular lean mass (ALM) excluding the bone mineral content (Goodpaster *et al*. 2006) was measured as the sum of the lean mass of arms and legs combined using standard regions of interest to determine arms and legs as previously reported (McPhee *et al*. 2013).

### EMG setup

The motor points of VL (proximal) and TA were identified by low‐intensity percutaneous electrical stimulations as previously described (Piasecki *et al*. 2016a,b). For VL, the motor point was located along the centre line of the muscle around 220 mm from the lateral femoral condyle. The TA motor point was located approximately 120 mm from the tibial head, over the muscle belly (Bowden & McNulty, 2012). The skin directly overlying the motor points was prepared by shaving away any hair and then cleansing with an alcohol swab.

The active surface EMG (sEMG) recording electrode (disposable self‐adhering Ag‐AgCl electrodes; 95 mm^2^, Ambu Neuroline, Baltorpbakken, Ballerup, Denmark) was placed over the motor point. The reference electrode was placed over the patella tendon for both muscles. Intramuscular EMG (iEMG) signals were recorded using disposable concentric needle electrodes (Model N53153; Teca, Hawthorne, NY, USA). A common ground electrode was used for sEMG and iEMG signals, which was placed over the patella for both muscles. The sEMG and iEMG signals were bandpass filtered at 5 Hz to 5 kHz and at 10 Hz to 10 kHz, respectively, using two CED 1902 amplifiers (Cambridge Electronics Design, Cambridge, UK). All signals were digitized with a CED Micro 1401 data acquisition unit (Cambridge Electronic Design). Sampling rates were 10 kHz for sEMG and 25 kHz for iEMG. Both EMG signals and the force signal were recorded and displayed in real‐time using Spike2 software (v8.01, Cambridge Electronics Design).

### Experimental procedures

To assess the knee extensor maximal voluntary contraction force (MVC), the participant's right leg was fastened to the force transducer 30 cm below the centre of the knee joint, with hip and knees flexed at 90°. For ankle dorsiflexion, participants sat with hips flexed at approximately 60° and both legs fully extended. The right foot was strapped to the force transducer with the ankle at 80° (Jones *et al*. 2009). Before testing MVC, participants performed a standardised warm up of several contractions, after which they were asked to perform a maximal effort lasting approximately 3 s with real‐time visual feedback and verbal encouragement provided. This was repeated twice more with short rest intervals between efforts. The highest of the three values was accepted as the MVC. In all cases, the knee extensors were first tested and participants then rested for a minimum of 10 min before the ankle dorsiflexors were tested.

In the VL, the maximal compound muscle action potential (CMAP) was obtained via percutaneous stimulation of the femoral nerve. The cathode was placed over the skin overlying the femoral nerve (approximately halfway between the anterior superior iliac spine and the pubic tubercle, proximal to the groin crease but distal to the inguinal nerve) and a carbon‐rubber anode electrode (Dermatrode self‐adhering electrode, 5.08 cm in diameter; Farmadomo Linde Homecare Benelux Bv, Leiden, The Netherlands) was placed over the skin overlying the gluteus muscle. In the TA, a bar electrode with the anode and cathode spaced 3 cm apart (Model MLADDF30; AD Instruments, Oxford, UK) was held over the common peroneal nerve around 5–10 mm distal of the fibular notch. Stimulation was applied using a manually triggered stimulator (model DS7AH; Digitimer, Welwyn Garden City, UK). Across a sequence of stimulations the current was increased incrementally until there was no further increase in CMAP. This generally occurred at 100–200 mA. To ensure supra‐maximal stimulation, the current was then increased by a further 30 mA.

After measuring the CMAP, the participant was asked to relax the muscle and an intramuscular needle electrode was inserted at approximately 60° angle to ensure the tip was beneath the active sEMG electrode and at approximately 2–3 cm depth. The needle position was adjusted to ensure the detection of motor unit potentials (MUPs) with sharp rise‐times during brief, low‐intensity voluntary contractions. The participant was then instructed to perform a muscle contraction to match as closely as possible a target line presented on a computer monitor set at 25% of the MVC. The contraction was held for 15 s, with real‐time visual feedback available throughout. Between four and six contractions were subsequently performed following the same procedures, with short rest intervals and the needle repositioned between contractions to sample from a range of depths and spatially distinct areas. This was done by combinations of rotating the needle 180° and withdrawing it by around 5 mm.

All EMG signals were recorded for offline analysis with decomposition‐based quantitative electromyography (DQEMG) (Stashuk, 1999). Our protocol required a minimum of 10 iEMG‐measured MUPs and the corresponding surface‐measured MUPs (sMUPs) to be recorded from each muscle for the data to be included in analysis. MUP area was defined as the total area within the MUP duration (Piasecki *et al*. 2016a,b).

### Motor unit number estimates: MUNE and iMUNE

The traditional motor unit number estimate (MUNE) methodology compares the average sMUP with the CMAP recorded following maximal electrical stimulation (Gooch *et al*. 2014; Piasecki *et al*. 2015). MUNE values were obtained using spike‐triggered ensemble‐averaging, in which MUP occurrence times identified from the iEMG signal were used to trigger sEMG signal epochs which were then ensemble‐averaged allowing sMUPs to be extracted (Brown *et al*. 1988). The sMUPs were then aligned based on onset time to create an ensemble‐averaged mean sMUP. The negative peak amplitude of the averaged sMUP was divided into the negative peak amplitude of the electrically evoked maximal CMAP to obtain an MUNE value. A further estimate of motor unit number, the intramuscular MUNE (iMUNE), was also made. This assumes that the MUP area recorded from iEMG is proportional to the CSA of the fibres of the active motor unit (Nandedkar *et al*. 1988a). Dividing the CSA of a muscle by the mean MUP area (cm^2^/mV/ms) provides a value proportional to the total number of MUs within that cross‐section of muscle (Piasecki *et al*. 2016a,b, 2018).

### Statistical analysis

Data are presented as mean (SD) where normally distributed or median (interquartile range, IQR) where not normally distributed. All VL QCSA data were converted to z‐scores relative to values in younger participants (where the values for young would be: mean = 0; SD = 1). Stages of sarcopenia in older participants were determined from QCSA threshold values in younger participants: sarcopenia was defined as axz‐score ≤ −2 to identify older men with muscle mass lower than values found within the 95% range of the normal distribution of young men, and corresponding knee extensor weakness. Pre‐sarcopenic men were identified as having muscle mass in the lower quartile of the distribution of young men (z‐score −1 to −1.99), and corresponding knee extensor weakness, but not meeting the criteria for sarcopenia. The non‐sarcopenic men were identified as older men who were not pre‐sarcopenic nor sarcopenic (z‐score > −0.99).

Where data were normally distributed, between‐group differences were compared using one‐way ANOVA. When significant differences were observed a Bonferroni *post‐hoc* test was performed. Where the data were not normally distributed, between‐group differences were compared using a Kruskal–Wallis test. When significant differences were observed, a Dunn–Bonferroni *post‐hoc* test was performed. Linear mixed models were used to assess group by muscle interactions, in which these factors were the fixed effects, and participants were random effects. Analysis was performed using SPSS Version 21 (SPSS, Chicago, IL, USA) software and *P *≤ 0.05 was considered statistically significant.

## Results

### Sarcopenia groups

**Figure 1:**
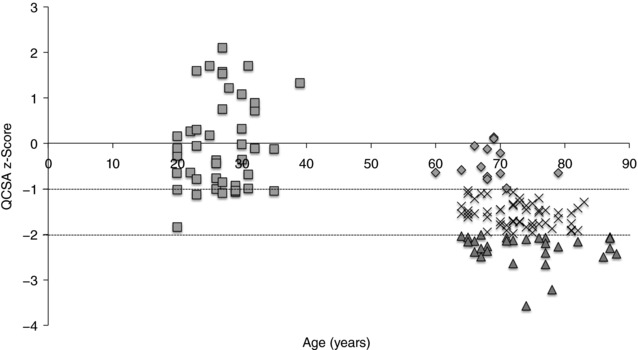
Quadriceps cross‐sectional area (QCSA) presented as z‐scores QCSA data were converted to z‐scores relative to the values of young men (young men's mean = 0; SD = 1). Young men shown as shaded squares; non‐sarcopenic older men are shown as shaded diamonds (z‐score ≥ −0.99); pre‐sarcopenic older men shown as crosses (z‐score between −1 and −1.99); sarcopenic older men shown as shaded triangles (z‐score ≤ −2). Dotted horizontal lines indicate −1 SD and −2 SD from the mean of younger participants.

Older participants were classified as non‐sarcopenic, pre‐sarcopenic or sarcopenic based on their QCSA z‐scores relative to values in younger people (Fig. 1). These groups had similar weight, BMI and body fat percentage. Younger men were taller than older men and this difference increased through the stages of sarcopenia. In the older men, the presence of sarcopenia was related to older age (Table 1).

**Table 1:** Participant characteristics and electrophysiological assessments

	Group				*P*‐values					
	Young (Y; *n* = 48)	Non‐sarcopenic (NS; *n* = 13)	Pre‐sarcopenic (PS; *n* = 53)	Sarcopenic (S; *n* = 29)	Y – NS	Y – PS	Y – S	NS – PS	NS – S	PS ‐ S
Quadriceps CSA (cm^2^)	91.0 (17.3)	83.5 (6.2)	64.0 (4.9)	50.9 (6.0)	0.790	**0.000**	**0.000**	**0.000**	**0.000**	**0.000**
**General**										
Age (years)	26.6 (4.9)	68.4 (4.3)	72.6 (5.2)	74.3 (7.9)	**0.000**	**0.000**	**0.000**	0.102	**0.011**	1.000
Height (m)	1.78 (0.06)	1.73 (0.06)	1.74 (0.06)	1.71 (0.07)	**0.013**	**0.001**	**0.000**	1.000	1.000	0.363
Weight (kg)	80.3 (14.8)	80.3 (11.2)	76.9 (12.7)	73.1 (13.4)	1.000	1.000	0.151	1.000	0.655	1.000
Body fat (%)	17.3 (8.8)	21.6 (10.7)	24.0 (10.1)	25.7 (8.3)	0.858	**0.003**	**0.002**	1.000	1.000	1.000
BMI (kg m^–2^)	25.1 (4.3)	26.9 (3.7)	25.3 (3.9)	24.8 (4.1)	0.888	1.000	1.000	1.000	0.767	1.000
**Components of sarcopenia**										
ALM/h^2^ (kg m^–2^)	8.54 (1.35)	8.43 (0.72)	7.45 (0.66)	6.66 (0.81)	0.578	**0.000**	**0.000**	**0.002**	**0.001**	**0.000**
QMuscle:FBone ratio	14.48 (2.28)	11.97 (1.85)	10.06 (1.12)	8.10 (1.46)	**0.000**	**0.000**	**0.000**	**0.006**	**0.000**	**0.000**
Knee extensor MVC (N)	588 (171)	389 (99)	361 (110)	302 (91)	**0.000**	**0.000**	**0.000**	0.494	**0.047**	**0.049**
TA CSA (cm^2^)	9.58 (1.73)	8.99 (1.26)	7.79 (2.05)	7.67 (1.59)	1.000	**0.000**	**0.000**	0.209	0.238	1.000
Ankle dorsiflexion MVC (N)	327 (110)	276 (63)	252 (60)	220 (82)	0.077	**0.001**	**0.000**	0.393	0.062	0.139
**Vastus lateralis electrophysiology**										
CMAP amplitude (mV)	11242 (3016)	8378 (2324)	7349 (2825)	7446 (2599)	**0.002**	**0.000**	**0.000**	0.254	0.335	0.881
sMUP amplitude (μV)	94.3 (60.7–118.9)	73.3 (49.1–95.0)	76.3 (54.1–116.9)	77.8 (44.4–117.3)	0.932	1.000	1.000	1.000	1.000	1.000
MUNE	115 (97–163)	105 (94–166)	92 (66‐133)	107 (71–142)	1.000	**0.007**	**0.070**	0.633	1.000	1.000
**Tibialis anterior electrophysiology**										
CMAP amplitude (mV)	11788 (3721)	5886 (2020)	6078 (2681)	6923 (3141)	**0.000**	**0.000**	**0.000**	1.000	1.000	1.000
sMUP amplitude (μV)	51.9 (36.6–67.0)	64.8 (50.5–128.1)	87.0 (49.6–118.7)	70.2 (49.5–90.8)	0.152	**0.000**	**0.035**	0.687	0.989	0.635
MUNE	239 (197–342)	77 (34–129)	71 (48–115)	99 (45–133)	**0.000**	**0.000**	**0.000**	0.981	0.743	0.649

Data are shown as mean (SD) where normally distributed, and as median (IQR) where not normally distributed. Abbreviations: CSA: cross‐sectional area; BMI: body mass index; ALM: appendicular lean mass; QMuscle:FBone: quadriceps cross‐sectional area to femur bone cross‐sectional area; MVC: maximum voluntary contraction; CMAP: compound muscle action potential; sMUP surface motor unit potential; MUNE: motor unit number estimate. *P* < 0.05 are displayed in bold.

### Muscle size and maximal force

As per the experimental design, muscle size decreased through the stages of non‐sarcopenic, pre‐sarcopenic and sarcopenic men (Table 1). Compared with young men, mean QCSA was 8% lower in the non‐sarcopenic men, 30% lower in the pre‐sarcopenic men and 44% lower in the sarcopenic men. Normalising QCSA to femur CSA to account for differences between people in body size produced similar results.

Height‐adjusted appendicular lean mass (ALM/h^2^) was similar for young and non‐sarcopenic old, but was 12% lower in pre‐sarcopenic men and 22% lower in sarcopenic men compared with the young. The mean value of 6.66 kg m^−2^ in the sarcopenic group was lower than the 7.26 kg m^−2^ cut‐off value commonly used to define sarcopenia (Cruz‐Jentoft *et al*. 2010).

Knee extensor MVC was 34, 39 and 49% lower for non‐sarcopenic old, pre‐sarcopenic and sarcopenic groups, respectively, compared with young men. Sarcopenic men had a lower MVC than pre‐sarcopenic and non‐sarcopenic old men (Table 1).

The ankle dorsiflexors were relatively well preserved compared to the age‐dependent differences seen in the knee extensors (4 group × 2 muscle interaction effect for CSA and MVC, both *P *< 0.001). TA CSA and dorsiflexion MVC were similar for young and non‐sarcopenic old men; but were 19 and 23%, respectively, lower in pre‐sarcopenic old, and 20 and 33%, respectively, lower in sarcopenic old than young men.

### Motor unit potential size

**Figure 2:**
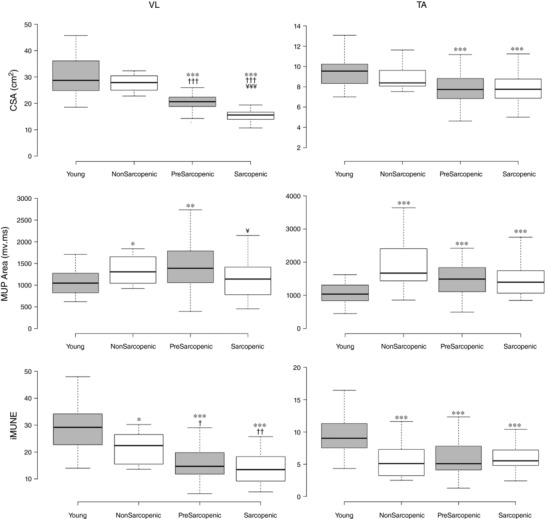
Muscle size, intramuscular MUP size and iMUNE values in different stages of sarcopenia Box plots indicate group median and upper and lower quartiles with Tukey whiskers. Comparison with young shown as ^*^ *P* < 0.05, ^**^ *P* < 0.01, ^***^ *P* < 0.001. Comparison with non‐sarcopenic shown as ^†^ *P* < 0.05, ^††^ *P* < 0.01, ^†††^ *P* < 0.001. Comparison with pre‐sarcopenic shown as ^¥^ *P* < 0.05, ^¥¥¥^ *P* < 0.001. VL: vastus lateralis; TA: tibialis anterior; CSA: cross‐sectional area; MUP: motor unit potential; iMUNE: intramuscular motor unit number estimate.

In the VL muscle, sMUPs did not differ between groups (Table 1), but intramuscularly measured MUPs were larger by 26 and 41%, respectively, in non‐sarcopenic and pre‐sarcopenic old than young men. MUPs were significantly smaller in sarcopenic compared with pre‐sarcopenic men (Fig. 2).

In the TA muscle, sMUPs and MUPs were larger in all older groups compared with young, with no difference between healthy old, pre‐sarcopenic or sarcopenic groups. There was a significant group × muscle interaction effect (4 groups, 2 muscles) for sMUPs (*P* = 0.001), but not for MUPs (*P* = 0.173).

Age was not significantly correlated with sMUP or intramuscular MUP size in VL or TA (all *r* < 0.02; *P* > 0.30) and adjusting for age in groups of older adults made no difference to the results of between‐group comparisons.

### Motor unit numbers

The MUNE value is derived from the ratio of CMAP/sMUP and is commonly used to indicate motor unit numbers in the TA. MUNE values for TA were 63–65% lower in older men compared with young men, with no difference between the non‐sarcopenic old, pre‐sarcopenic and sarcopenic groups (Table 1).

The iMUNE method provides values proportional to motor unit number after accounting for muscle size. TA iMUNE was 48% lower in all older groups compared with young men, with no difference between non‐sarcopenic old, pre‐sarcopenic and sarcopenic groups (Fig. 2). VL iMUNE was 33, 47 and 50% lower in non‐sarcopenic old, pre‐sarcopenic and sarcopenic, respectively, compared with young. Values in pre‐sarcopenic and sarcopenic groups were similar, and both were significantly lower than the non‐sarcopenic old. A significant group × muscle interaction (4 groups × 2 muscle) showed the difference between groups in iMUNE was greater in TA than in VL (*P* < 0.001).

When looking at groups of older men, age was not significantly correlated with iMUNE in either VL or TA (all *r* < 0.02; *P* > 0.50), and adjusting for age in groups of older adults made no difference to the results of between‐group comparisons.

## Discussion

Our study is the first to directly compare motor unit number and size estimates in adults with different levels of sarcopenia. The results show that loss of motor units occurs relatively early during ageing, exceeds the loss of muscle mass and precedes sarcopenia. The surviving motor units were enlarged in the non‐sarcopenic and pre‐sarcopenic old, but not in the sarcopenic old. These findings suggest that a failure to expand the motor unit size distinguishes sarcopenic from pre‐sarcopenic muscles.

A recent study compared TA motor unit numbers in groups described as pre‐sarcopenic, sarcopenic and severe sarcopenic (Gilmore *et al*. 2017). In agreement with our findings for TA, they found that motor unit numbers were similar across groups. However, the small sample size (*n* = 7 sarcopenic; *n* = 5 severely sarcopenic), and the fact that muscle size did not differ significantly between the groups described as pre‐sarcopenic, sarcopenic and severe sarcopenic limits the conclusions of this study. Definitions of sarcopenia have evolved over the past two decades, but low muscle mass is the definitive criterion (Cruz‐Jentoft *et al*. 2010).

In the present study, both the MRI and the DXA measurements clearly demonstrated a progressive decline of muscle mass across groups (Fig. 1, Table 1). The 26 sarcopenic men would be classified as such by all commonly used definitions due to their low knee extensor strength, and the average ALM/h^2^ of 6.66 kg m^−2^ was well below accepted cut‐offs, such as the 7.26 kg m^−2^ recommended by a European Working Group (Cruz‐Jentoft *et al*. 2010).

### Motor unit number estimates

Recent technical advances enable enhanced decomposition of intramuscular EMG signals into MUPs and identification of the corresponding sMUPs (Parsaei *et al*. 2012; Parsaei & Stashuk, 2013), proportional to motor unit size (Nandedkar *et al*. 1988a,b). Normalising the ensemble‐averaged sMUP to the CMAP provides an estimate of motor unit number by asking, how many motor units fit into the CMAP? (Bromberg, 2007; Gooch *et al*. 2014). A technique was adapted for the larger VL (also relevant to the TA) to reduce effects of EMG signal attenuation from deeper muscle regions (Muceli *et al*. 2015). It normalises the motor unit size to the muscle cross‐section (known as iMUNE; Piasecki *et al*. 2018) to ask simply, how many motor units fit into the muscle cross‐section? Both techniques suggest that the group of non‐sarcopenic men (mean age 68 years) with similar size muscles to young men had approximately 40% fewer TA motor units than young, and the iMUNE estimated approximately 30% fewer VL motor units than young (Table 1, Fig. 2).

Our hypothesis that motor unit numbers would progressively decrease from young adults through to healthy old, pre‐sarcopenic and sarcopenic men was not supported in the TA. TA motor unit numbers were already low in the non‐sarcopenic group and remained at similar levels in pre‐sarcopenic and sarcopenic men (Table 1). This hypothesis was based on the assumption that muscle size would decrease with increasing sarcopenia in older participants, but this was not the case for TA (Fig. 2). In line with previous reports (Abe *et al*. 2011; Pannerec *et al*. 2016), the TA was only 10–15% smaller in older participants compared with the young, which was significantly less than the 30–44% quadriceps atrophy affecting pre‐sarcopenic and sarcopenic men (Table 1, Fig. 2).

The VL iMUNE values were highest in the young, intermediate in the non‐sarcopenic old and lowest in the pre‐sarcopenic and sarcopenic men (Fig. 2). The younger average age of the non‐sarcopenic men might have been a reason for their higher VL iMUNE and muscle mass when compared to sarcopenic men (McNeil *et al*. 2005). However, we found that when looking at all older men together, there was no relationship between age and MUNE, iMUNE or muscle size, so advancing older age per se does not appear to be the critical factor determining limb motor unit numbers or sarcopenia status.

Our observation that TA had considerably fewer motor units in older men but muscle size was relatively well preserved, and that pre‐sarcopenic and sarcopenic groups had different muscle mass but similar motor unit numbers in VL, does not support our original study hypothesis. Rather, these data suggest that loss of muscle mass does not occur in direct proportion to declining motor unit numbers. The observation that the size of motor units differed across older groups suggests that this factor also contributes to muscle lost during ageing.

### Motor unit size estimates

Previous work from several sources indicates that muscle size can be preserved despite the considerable loss of motor units if the surviving units expand by sprouting new branches to innervate the denervated muscle fibres (Deschenes, 2011; Kung *et al*. 2014). Because loss of motor units is age‐related, this process leads to larger motor units in older compared to young people. A summary of results from different studies found 49% larger motor unit potentials in older compared with younger people, but there was large heterogeneity in findings between muscles and between studies (Dalton *et al*. 2008; Hourigan *et al*. 2015; Piasecki *et al*. 2016a,b). In the present study, we observed that in non‐sarcopenic and pre‐sarcopenic elderly men, a doubling of motor unit size in TA and a 26–42% higher motor unit size in VL (Fig. 2) was not associated with preservation of muscle mass (10–15% lower in TA and 30–44% lower in quadriceps; Table 1). A failure to rescue some fibres leading to fewer fibres in older than younger men, alongside age‐related muscle fibre atrophy (primarily affecting type 2 fibres) (Lexell *et al*. 1988; Barnouin *et al*. 2017), accounts for the net loss of muscle mass.

Crucially, our results show for the first time that sarcopenic muscles do not follow the expected trend towards increasing motor unit size. Instead, motor units were significantly smaller in sarcopenic than in pre‐sarcopenic men (Fig. 2). There are two probable explanations for this. First, the average muscle fibre CSAs of sarcopenic individuals may be relatively small, similar to those found in very old adults (Scelsi *et al*. 1980) Secondly, sarcopenic muscle may experience a failure of reinnervation, similar to muscle of octogenarians (Spendiff *et al*. 2016), which would precipitate myofibre loss, leaving motor units with lower innervation ratios and smaller motor unit potentials. Similar characteristics are seen in severely atrophied rodent muscles with increased numbers of very small fibres expressing markers of denervation (Rowan *et al*. 2012), probably due to a failure of reinnervation (Aare *et al*. 2016).

Overall, these findings have important implications for the progression of sarcopenia and the prospects of reversing this condition. When motor neurons or muscle fibres are lost, it is possible that they can never be replaced and this could limit the efficacy of any therapeutic interventions and, consequently, more emphasis should be placed on the prevention of sarcopenia. The pathophysiological processes determining the fate of motor neurons and denervated fibres remain unknown and this is clearly an important area for future research. Some results suggest that long‐term exercise training might prevent the loss of motor units (Power *et al*. 2010, 2016), but others show no obvious sparing of motor unit numbers with life‐long exercise (Piasecki *et al*. 2016a).

### Strengths and limitations

Our study has several strengths: (a) it is the first to directly compare motor unit number and size in adults with different levels of sarcopenia; (b) the largest sample size of any studies of motor units in older age gives higher statistical power to identify important relationships when compared to previous studies; and (c) the latest iEMG and decomposition techniques were used to determine MUPs and accurate imaging including MRI and DEXA scanning enabled accurate determination of muscle size and thus sarcopenic status.

There are several limitations. (a) As in all clinical studies, direct motor unit counts were not possible (Piasecki *et al*. 2015; Hepple & Rice, 2016), so indirect estimates from intramuscular EMG were used to derive values proportional to motor unit numbers. These techniques are very well established and take the MUP size as representative of motor unit size (Nandedkar *et al*. 1988a). Normalisation to the CMAP for the MUNE, or muscle CSA for the iMUNE, is straightforward. (b) We were only able to confidently decompose individual motor units from EMG signals during moderate‐intensity contractions. According to the ‘size principle’ (Henneman *et al*. 1965), the larger, high‐threshold motor units may not have been active and may have differed between groups. However, this has the added advantage that the active motor units would be mainly composed of the earlier recruited type 1 muscle fibres, and because old and young adults have similar type 1 fibre CSA (Lexell *et al*. 1988; Barnouin *et al*. 2017) we can be confident that the increase in MUPs from young to non‐sarcopenic and pre‐sarcopenic men is due to increased motor unit size rather than increased fibre CSA or circumference.

### Conclusions

We have shown that motor unit losses occur relatively early during the ageing process and this contributes to the sarcopenic condition. The data suggest that the expansion of surviving motor units provides a regulatory mechanism to preserve muscle mass. Importantly, what distinguished sarcopenic from pre‐sarcopenic men was a failure to expand motor unit size. Overall, our results suggest that both the loss of motor neurons per se and the collateral sprouting from surviving motor nerve branches to reinnervate muscle fibres of defunct motor units are critical factors determining the progression of sarcopenia.

## Additional information

### Competing interests

The authors declare that they have no competing interests.

### Author contributions

All authors contributed to the design of the work. MP, AL, JP, AS and JSM contributed to acquisition of the data. All authors contributed to analysis and interpretation of the data. MP and JSM drafted the manuscript. AL, JP, DS, AS, MR and DJ provided comments and approved the final submission. All authors agree to be accountable for all aspects of the work.

### Funding

This work was supported by funding from the UK Medical Research Council as part of the Life Long health and Wellbeing initiative (MR/K025252/1).
